# The Lady Priestley Lecture

**Published:** 1920-05-01

**Authors:** 


### ON PREVENTIVE MEDICINE.

### The Lady Priestley Lecture.

The large Barnes Hall of the Royal Society of Medicine was filled by a very appreciative audience on Thursday, April 22, to hear Sir George Newman deliver the tenth memorial lecture in memory of the late Lady Priestley, under the auspices of the National Health Society. H.R.H. Princess Christian was present, and graciously presented to successful candidates at the moment in London the certificates they had gained at the examination to qualify as health visitors.

The chair was occupied by Sir James Crichton-Brown, F.R.S., the Chairman of the Society, whose introduction of the lecturer was brief but happy, setting out in a few masterly sentences the history of the National Health Society's efforts of the foundation of the Lectureship, and of Sir George Newman's earlier persistent work in the cause of public health before he achieved his present distinguished position in the Ministry of Health.

### The Lecture.

Sir George Newman's title was "The Place of Public Opinion in Preventive Medicine," and the lecture was perhaps the best summary of the position and potentialities of public-health effort that we have heard. It was exceedingly well delivered, and was frequently punctuated by applause. He declared that the English people are suffering from impaired physique; in 1914, of the children who left school in the healthiest districts 10 per cent. had grave physical disability, 20 per cent. had defective vision, while upwards of 60 per cent. suffered from severe dental caries. More than half the insured people in England and Wales claimed and received medical treatment every year. Ten years of medical inspection in elementary schools had shown that a million children of school age were so defective as to be unable to derive reasonable benefit from their schooling. In this country every week there were 1,000 funerals of persons who had died of tuberculosis, 1,200 of dead infants, 800 due to death from cancer, while in 1918 100,000 persons died of influenza. In employment there were fourteen million weeks of lost time, equalling 270,000 years per annum, most of it due to preventable sickness!

### "The Man in the Street."

Many more equally eloquent facts were adduced, but Sir George's main theme was the function of the public in improving all this. Some kind of public opinion had existed from earliest times. Sir Robert Peel defined it as "a great compound of folly, weakness, prejudice, wrong feeling, right feeling, obstinacy, and newspaper paragraphs." Some of this, said the lecturer, was true to-day. The "man in the street" was the ultimate master. The coming of the Ministry of Health meant a new sort of attack on the strongholds of disease; increased State intervention and organisation and a bolder policy. But an even more important factor was an educated community and an enlightened public opinion. It had been well said that only an educated people was an effective and healthy people, and here technical instruction in hygiene was not meant, but an informed humanism which welcomed and understood the growth of medicine, accepting its results boldly and gladly on behalf of mankind. Sir George proceeded to emphasise the importance of nutrition, fresh air, and exercise, with as ancillaries cleanliness and warmth. Healthy and complete nutrition was much more comprehensive than the mere filling of the stomach; it connoted a healthy body in all respects, a brain and nervous system in tone, a healthy muscular and digestive system, circulation of blood and lymph. But the dietetic conditions of the great mass of the workers meant a tale of bread and beer, tea and pickles, canned meat and cakes. Appetising cookery and freshly prepared food were often absent. Only rarely was poverty the reason of this lack; it lay in ignorance concerning the right food to buy and how to cook it. And in the matter of ventilation of home and workshop, mostly the means for securing that were already there; what was lacking was the maintenance of ventilation by the individual, the desire to do so being based upon his appreciation of its need.

### Games and Recreation.

The value of games and recreation for all the people was eloquently pleaded. It was not enough that 40,000 youths should watch football matches or boxing bouts; the need was for all to participate in playing the game, and well beyond the period of youth. Nor, in this connection, must women, the source of the race, be forgotten. He insisted on the great protective power against disease of a sound, vigorous body, pointing out how great a reserve of disease-resisting capacity such an organism possesses by the production of anti-bodies. The thoughtless dissemination of disease germs by sufferers from colds produced greatly lessened results in those who maintained sound bodily vigour. But hygiene could become an expression of the national life only if the people consented and were willing to advocate and carry out its reforms; without this support mere legislation would prove abortive.

Sir George would give that instruction to the whole child and adolescent population; the means of healthy living must be pressed home every week in every school in the land, and a new opportunity for this was before us in the Continuation Schools, attendance at which was now compulsory. But even wider methods of propaganda were needed; the services of all who know must, he said, be impressed for and on behalf of those who do not know. All doctors, nurses, midwives, health visitors, sanitary inspectors and welfare workers should

**On Preventive Medicine**—(*continued*)

be missionaries of hygiene. Why not have a "Health Day"? asked Sir George. The Press had done much to further the cause of the national health, but he thought it might very well do more. In this connection he had a flattering word for the medical journals. Lastly, he thought the Government itself could not be absolved from its share of responsibility in begetting a wise public opinion on health matters. During the earlier part of his thesis he had particularised the work of the R.A.M.C. and its results, as well as the value of research. The present prevalence of venereal disease he regarded as a stain upon our civilisation. Education, enlightenment, early skilled treatment and a wholesome public opinion would prove to be the great ultimate cleansers of our homes, thus bringing nearer the great objective of preventive medicine: to make human life better, larger, more capable and useful, happier, and to prolong our days. In a noble peroration Sir George declared that the spirit and character of her public opinion was one of the enduring glories of England: could it be devoted, he asked, to a nobler cause than the physical and mental health of her people?

Thanks.

On the proposition of Mr. Adrian Pollock (Chamberlain of the City of London) a cordial vote of thanks to H.R.H. Princess Christian for kindly presenting the certificates was carried by acclamation. The thanks of the meeting were also warmly accorded to Sir George Newman for his lecture, on the motion of Professor Kenwood, seconded by Dr. S. Higgins, Medical Officer of Health for St. Pancras.

Sir James Crichton-Browne was thanked for his services as Chairman of the Society and for presiding over this meeting, on the proposition of Sir Ray Lankester, K.C.B., seconded by General Sir John Goodwin. A similar vote was passed to the Council of the Royal Society of Medicine for the use of the Hall for the meeting.

